# Form Follows Function: Structural and Catalytic Variation in the Class A Flavoprotein Monooxygenases

**DOI:** 10.3390/ijms131215601

**Published:** 2012-11-23

**Authors:** Karen Crozier-Reabe, Graham R. Moran

**Affiliations:** Department of Chemistry and Biochemistry, University of Wisconsin-Milwaukee, 3210 N. Cramer Street, Milwaukee, Wisconsin 53211-3029, WI, USA

**Keywords:** monooxygenase, flavin, flavoprotein, molecular oxygen, oxygenase, monooxygenase

## Abstract

Flavoprotein monooxygenases (FPMOs) exhibit an array of mechanistic solutions to a common chemical objective; the monooxygenation of a target substrate. Each FPMO efficiently couples reduction of a flavin cofactor by NAD(P)H to oxygenation of the target substrate via a (hydro)peroxyflavin intermediate. This purpose of this review is to describe in detail the Class A flavoprotein hydroxylases (FPMO) in the context of the other FPMO classes (B–F). Both one and two component FPMOs are found in nature. Two-component enzymes require, in addition to the monooxygenase, the involvement of a reductase that first catalyzes the reduction of the flavin by NAD(P)H. The Class A and B FPMOs are single-component and manage to orchestrate the same net reaction within a single peptide. The Class A enzymes have, by some considerable margin, the most complete research record. These enzymes use choreographed movements of the flavin ring that facilitate access of the organic substrates to the active site, provide a means for interaction of NADPH with the flavin, offer a mechanism to sequester the dioxygen reduction chemistry from solvent and a means to release the product. The majority of the discrete catalytic events of the catalytic cycle can be observed directly in exquisite detail using spectrophotometric kinetic methods and many of the key mechanistic conclusions are further supported by structural data. This review attempts to compile each of the key observations made for both paradigm and newly discovered examples of Class A FPMOs into a complete catalytic description of one enzymatic turnover.

## 1. Introduction

Common activating substitutents of aromatic molecules such as a hydroxyl or an amino group, facilitate catabolic degradation by enhancing nucleophilicity. The catabolism of such molecules provides access to considerable pool of energy for living organisms. For example, phenolics are second only to carbohydrates in their abundance nature as they are liberated from lignin, a structural polymer of all woody plants [1,2]. An increase in activated aromatic molecules in the environment has also resulted from industrial activity [3]. Mesophilic microorganisms have been shown to aerobically catabolize both the natural and xenobiotic aromatic compounds by first further activating the ring by the addition of a hydroxyl group [4]. The enzymes that carry out this early catabolic step are often flavoprotein monooxygenases (FPMOs). Metal ion dependent dioxygenases can then cleave the resulting catechol or hydroquinone rings and the aliphatic products that result are then cleaved further to enter primary energy yielding metabolic pathways [5]. Though the core or original function of flavoprotein hydroxylases is catabolism, they are also found to perform tailoring functions in biosynthetic pathways [6,7]. While the majority of the known FPMOs hydroxylate phenolic substrates (referred to hereafter as the target substrate) there are others whose target substrates have an amino substituent or are non-aromatic or lack an activating substituent entirely [8–13].

The source of the oxygen atom that is added in the form of a hydroxyl is molecular oxygen. All FPMOs use the functionality of the reduced flavin isoalloxazine ring system to mediate the acquisition/reduction/activation of dioxygen. As such FPMOs most often require an external electron source such as reduced nicotinamide to first reduce the flavin by two electrons. Those enzymes that require a reductant are known as external FPMOs.

An extraordinary wealth of data describes the function, structure and catalytic chemistry of the FPMOs and recent reviews have summarized key aspects of the lineage and chemistry of these catalysts [14–16]. However, it is clear that the basis of understanding of these enzymes has come from the study of only a few highly tractable examples. This review catalogs structural and mechanistic variation amongst both paradigm and more peripheral members of the Class A FPMO enzymes. It is not intended to be a chronology of the research of these enzymes and focuses instead on the more current ideas that have emerged regarding how enzyme structure directs the reaction forward.

### 1.1. The Flavin Cofactor

**Figure 1:**
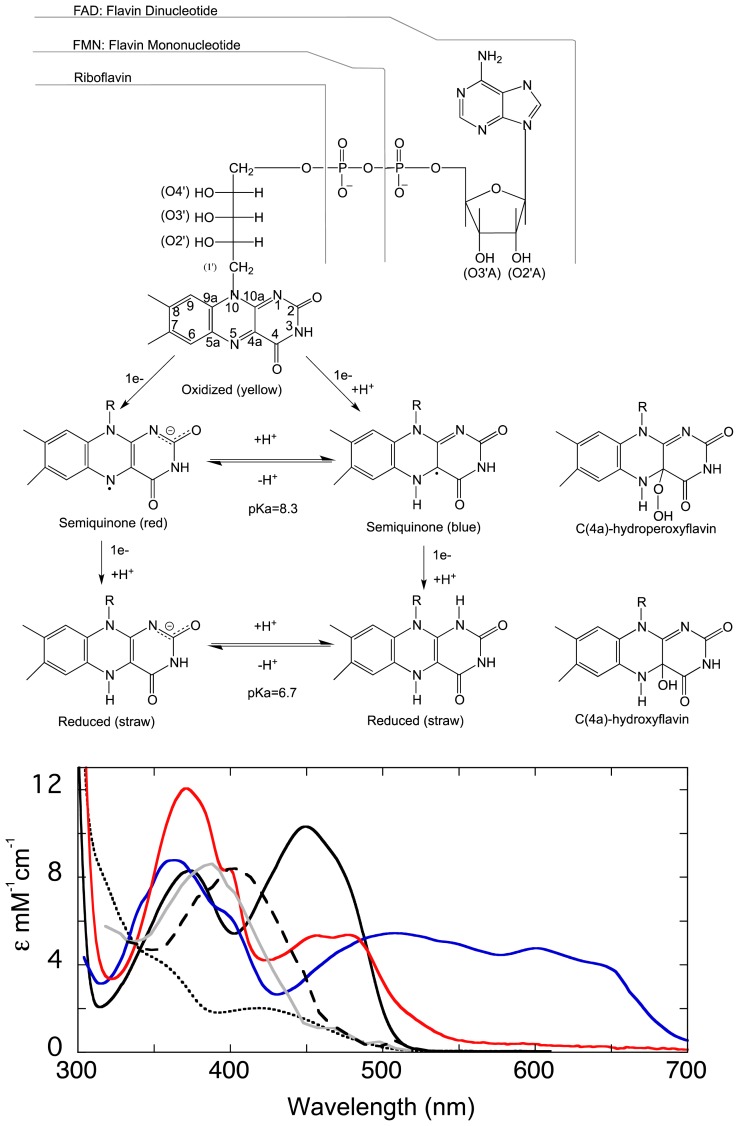
Flavin structure, redox states, oxygen adduct structures and characteristic spectra. All structures are shown from the *re*-face. The spectra shown are representative examples for the: (–––) oxidized flavin, (----) reduced flavin, (red) red semiquinone, (blue) blue semiquinone, (gray) *C*(4a)-hydroperoxyflavin, (— —) *C*(4a)-hydroxyflavin.

Flavin is requisite to all life, approximately 1%–3% of all genes encode FAD- and FMN-binding proteins [17–19]. Although flavin is required by all organisms, animals lack the ability to synthesize it and must obtain it from their diet. Flavins are found to exist in a number of forms in nature, the most common being flavin adenine dinucleotide (FAD), flavin mononucleotide (FMN), and riboflavin (Figure 1). FPMOs typically use FAD though FMN is also observed. FAD and FMN are derived from riboflavin (vitamin B_2_), which is biosynthesized from GTP and ribulose 5-phosphate [20]. FAD is a dinucleotide and consists of a tricyclic isoalloxazine base bonded via a ribityl phosphate that is in turn linked by a phosphodiester bond to an adenosine monophosphate (Figure 1). In most cases it is the adenosine monophoshate portion that anchors the molecule to the protein and the flavin generally remains associated throughout catalysis. Most flavo-proteins associate with the flavin prosthetic group tightly and form 5–15 hydrogen-bonding, charge pairing, and van der Waals interactions (below). A grouping of secondary structural elements comprise this nucleotide binding pocket that are known collectively as the Rossmann fold, which is common to but not exclusively observed in the FPMOs [21].

The isoalloxazine ring of the flavin cofactor is a highly versatile redox moiety that, contingent on its oxidation state, can receive or donate electrons one or two at a time. The two-electron or fully reduced form of the flavin can therefore mediate in the two single electron transfers required to reduce the triplet ground-state dioxygen molecule to the peroxo species. This process in the absence of such a mediator would be spin-forbidden. While other cofactors such as pyridoxal phosphate or biopterin offer signature spectra that are readily identifiable as discrete chemical states, the flavin is the most spectrophotometrically diagnostic organic cofactor. The isoalloxazine has three oxidation states: the bright yellow oxidized form, the one-electron reduced or semiquinone form, that is observed either as a neutral blue molecule or as a red anionic species, and the pale straw-colored two-electron reduced hydroquinone that also has neutral and ionic forms. The oxidized, reduced and oxygen adduct forms are observed with the FPMOs (Figure 1) [22].

The neutral and anionic semiquinone species are more rarely observed, stabilized for extended periods in only a few oxidase, electron transferase and lyase enzymes [23–26]. The unique spectrophotometric signature of each oxidation and protonation state and a number of highly recognizable flavin adducts facilitates observation and recognition of discrete events during flavoenzyme catalysis [27,28]. In addition, the oxidized and oxo-adduct forms of the flavin have unique fluorescence emission spectra that offer another physical signal to observe processes such as ligand binding and catalyisis. The wealth of identifiable spectrophotometric changes permit the researcher a window into enzyme mechanism that is unrivaled by any other organic cofactor or substrate [29,30].

The chemical properties of the flavin isoalloxazine are highly sensitive to the local environment. Flavo-enzymes manipulate the character of the isoalloxazine ring system for specific reactions. An illustrative example is that the reduction potential of the isoalloxazine can vary widely. In solution at pH 7.0, the two electron reduction potential of free flavin is about −210 mV [31,32]. However, a range of potentials are observed when bound to specific proteins where the isoalloxazine reduction potential can vary from −495 mV for the semiquinone/reduced couple in flavodoxin to +40 mV for the two electron oxidized/reduced couple in thiamin dehydrogenase [33,34]. The reduction potentials of the FPMOs, however, tend to remain closer to that of free flavin at about −180 to −200 mV [35–37].

### 1.2. The FPMO Classes

An oxidoreductase that catalyzes the incorporation of one or more atoms of molecular oxygen into a target substrate is referred to as an oxygenase [38,39]. All oxygenases require a cofactor in order to activate molecular oxygen. Flavin-dependent monooxygenase are exceptional in their ability to sequentially deliver electrons in the reduction of molecular oxygen. Oxygenases more typically require the participation of redox-active metal ions to facilitate such reactions. There are five primary classes of redox active flavoenzymes: disulfide oxidoreductases, dehydrogenases, oxidases, dioxygenases, and monooxygenases. FPMOs incorporate one atom of molecular oxygen onto their target substrates and the second atom is fully reduced to form water. Two classes of flavoprotein monooxygenases are known, the internal and the external monooxygenases. The internal monooxygenases are quite rare [32]. They catalyze a variation of flavoprotein oxidase chemistry and initiate their reactions by oxidizing the target substrate by two electrons and then carry out hydroxylation and oxidative decarboxylation [40]. The target substrate thus donates in total four electrons to bring both atoms of the dioxygen molecule to hydroxyl oxidation state. As a rare exception among the FPMOs, these enzymes will not be described further in this review and the abbrevaition FPMO will, unless stated, refer only to those enzymes that use pyridine nucleotide as a reductant. The external FPMOs reduce molecular oxygen by four electrons, two electrons from an external cosubstrate (NAD(P)H) delivered in the form of a hydride are transferred to the flavin and two additional electrons are supplied from the target substrate.

**Scheme 1:**
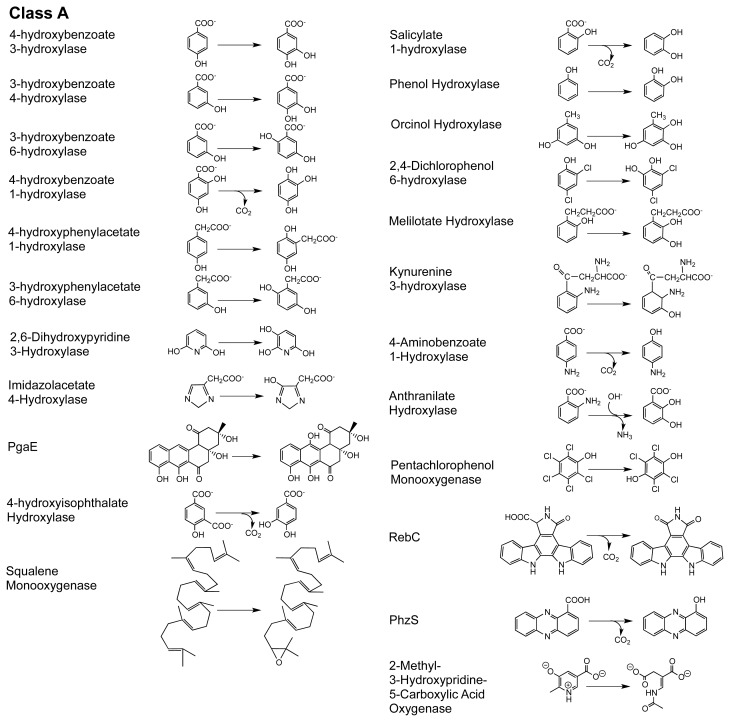

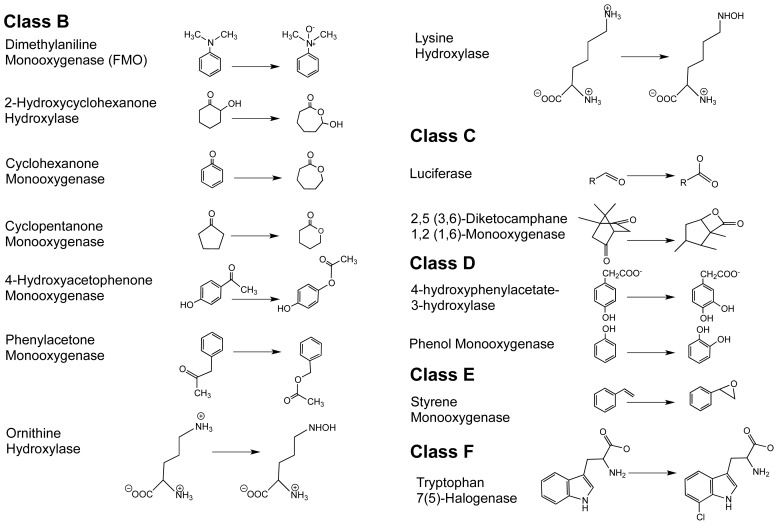
The reactions of the external flavoprotein monooxygenases. While the majority of known Class A activities are depicted, only representative reactions for Classes B–F are shown. Linear, left to right arrows represent the oxidation of NAD(P)H, the consumption of dioxygen and the liberation of water. The delineation into alphabetic groupings is based on the classification of van Berkel *et al.*[14].

**Scheme 2:**
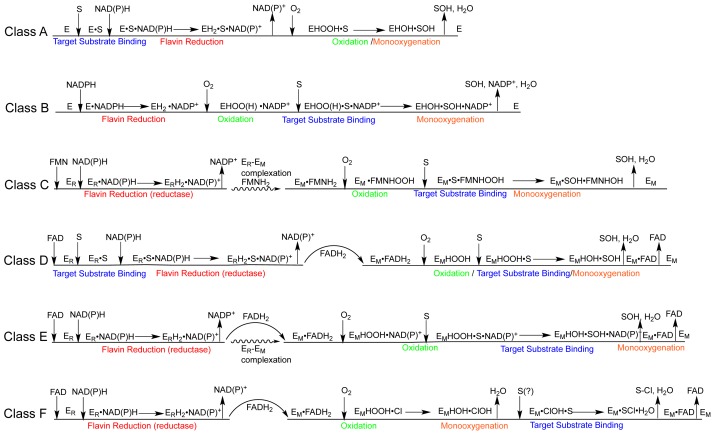
The Proposed Kinetic Mechanisms of the Flavoprotein Monooxygenases (FPMOs) Representative reaction schemes for each class of FPMO enzyme are shown. Within any one class, variation in the order of events for flavin reduction or target substrate monooxygenation may occur for individual cases. Substrates and products are shown to enter and leave the reaction by the use of vertical arrows. E_R_ refers to the reductase component of a two component system, while E_M_ indicates the monooxygenase component. When the reduced flavin is transferred between the reductase and the monooxygenase by diffusion the transfer is depicted as an arcing arrow. When the reduced flavin is channeled across a complex of the reductase and the monooxygenase it is depicted as an oscillating arrow. S refers to the target substrate.

Van Berkel delineated six classes of external flavoprotein monooxygenase on the basis of amino acid sequence, tertiary structure and cofactor preference [14] (Schemes 1 and 2). It is also possible to delineate these classes to some extent by mechanism or by the type of target substrate that is oxygenated. As new class members are discovered, exceptions in the sequence of catalytic steps are observed that can blur the mechanistic distinctions between classes. What is clear, however, is the overall catalytic strategy of the entire family. The mechanism of any one member is compiled from a common and seemingly modular set of reactions. Scheme 2 is a summary of the commonly observed order of events for each class. The common chemistries of the entire family are: reduction of the flavin using NAD(P)H, stabilization of a peroxy form of the flavin that is formed from the reaction of the reduced flavin with dioxygen, the use of this intermediate to carry out the oxygenation of a generally activated target substrate and the elimination of the second oxygen atom derived from dioxygen from the residual hydroxy-flavin as a molecule of water.

Class A flavoprotein monooxygenases are the focus of this review. These are single component enzymes that have substrate binding and the reductive and oxidative half reactions orchestrated by a single peptide and use FAD that is bound tightly and non-covalently. The typically aromatic target substrate and NADPH are first to add and formally bind randomly, however, the binding of the target substrate greatly stimulates the reduction of the flavin by NAD(P)H [41]. NADP^+^ must then dissociate so that molecular oxygen can gain access to the flavin and form a reactive C4a-hydroperoxyflavin that, compared to those formed on enzymes in classes B–F, is somewhat labile. This species acts as a weak electrophile, typically hydroxylating an aromatic substrate *ortho* or *para* to an existing activating substituent. The inability to hyper-stabilize the peroxy form of the flavin in the absence of the target substrate is characteristic of the Class A FPMOs [42].

Class B flavoprotein monooxygenases are also single component enzymes that may or may not bind FAD tightly prior to turnover [43–46]. They are distinguished from Class A by the order in which substrates add to the enzyme (Scheme 2). Class B enzymes undergo the reductive half of the catalytic cycle and retain the oxidized nicotinamide throughout catalysis. The reduced enzyme reacts with dioxygen to form the hydroperoxyflavin. It is typically at this stage that the binding of the target substrate occurs though it is occasionally acquired prior steps along with the pyridine nucleotide substrate [43,47,48]. It is thought that NADP^+^ is retained throughout catalysis to help stabilize the reactive flavin-oxo adduct and thus it follows that the final and rate limiting step is the dissociation of NADP^+^[49]. Recent structures of ornithine hydroxylase and phenylacetone monooxygenase confirm this and give a detailed description of the relative locations of FAD with respect to NADP^+^ and the target substrate and product [48,50,51].

Class C–E flavoprotein monooxygenases exhibit gross structural differences and unique mechanisms [14]. These enzymes are predominantly two-component systems having a reductase and a monooxygenase that are coded by separate genes though occasionally the two genes are fused, tethering the reductase and monooxygenase activities [16]. The reductase catalyzes the reduction of the flavin (by NAD(P)H) that is destined to ultimately reside in the monooxygenase and react with dioxygen. It is more common to observe that the apo-reductase will bind both NAD(P)H and flavin as substrates and catalyze hydride transfer between them [52]. There are exceptions, the reductase of phenol hydroxylase A2 (class D Schemes 1 and 2) has a tightly bound flavin that mediates in a double displacement where NADPH first reduces the bound flavin, then the oxidized nicotinamide dissociates ahead of the binding of another flavin that, once reduced, will be shuttled to the monooxygenase [53]. In an unexpected correlation with the Class A enzymes, the binding of the target substrate to the reductase in the class D enzyme, 4-hydroxyphenylacetate 3-hydroxylase can stimulate the rate of reduction by 1–2 orders of magnitude despite that hydroxylation will occur on the monooxygenase component [54]. Multicomponent FPMOs have developed a number of strategies to reduce the flavin cofactor and then shuttle it from the reductase to the monooxygenase. The reductases typically show higher affinity for oxidized flavin and lessened affinity for the reduced form and the reverse is true at the monooxygenase [55]. However, for those systems in which it has been measured the monooxygenase shows the greatest difference in binding affinity for the oxidized and reduced forms (up to 4 orders of magnitude) and as such provides the thermodynamic driving force that maintains efficiency within the system. Shuttling can result from either the dihydroflavin being released from the reductase into solution and bound both rapidly and tightly by the monooxygenase or when it is exchanged in a complex of the two components that channels the reduced flavin between them [56]. One could readily imagine a continuum of two component systems that rely to a greater degree or less on one or both strategies [57,58]. In either case, once the flavin is bound by the monooxygenase component it reacts with molecular oxygen before association of the target substrate and it has been shown that the target substrate preferentially adds to the (hydro)peroxy form of the enzyme [59]. Once associated, the target substrate is oxygenated leaving only the elimination of water from the hydroxyflavin and the release of the product along with the now oxidized flavin to regenerate the free monooxygenase.

Class F is distinct from all other external flavoprotein monooxygenases in that, the target substrate is not hydroxylated but instead halogenated indirectly via a hydroxylation reaction with a bound halide. There is some debate with regard to the enzyme’s reaction mechanism; however, it has been proposed that the *C*4a-hydroperoxyflavin hydroxylates a proximally bound chloride ion. The hydroxylation product, hypochlorous acid, is retained and halogenates an aromatic target substrate that is localized in an adjoining binding pocket [60–64].

## 2. The Class A FPMOs

Class A is the largest of the FPMO subgroups and these catalysts almost exclusively hydroxylate aromatic rings, generally resulting in aromatic or occasionally ring cleaved products [15,65] (Scheme 1). A select number of highly tractable Class A enzymes have been studied for more than forty years and these enzymes have become the paradigm to which all other flavoprotein monooxygenases are compared. The preponderance of what is known of this class has come from studies of *para*-hydroxybenzoate hydroxylase (PHBH) and to a lesser extent phenol hydroxylase (PHHY). While many of the same or similar observations have been made for other members of this class, and for other classes, for none is the understanding of the discrete events of catalysis as complete as it is for PHBH and PHHY. On this basis and to provide a context for the integrated structural description below, the general understanding of catalysis by the Class A enzymes is as follows. Class A enzymes bind FAD non-covalently and with high affinity in an elongated conformation [21]. The isoalloxazine ring can change position during catalysis, pivoting at the ribityl C(2) so that it can swing 7–8 angstroms from a largely internal position within the protein to the exposed solvent edge and also to intermediate positions between these extremes [66–69]. In the resting enzyme the flavin ring is thought to reside internal to the structure in an *open-in* position [70,71]. This position allows surface residues to direct the target substrate through a channel into a binding pocket that secures the ligand by hydrogen bonds and/or charge pairing interactions [67,72]. Several positively charged residues at the solvent interface bind NADPH and this association induces the flavin to pivot to the most exposed *out* conformation to undergo reduction [72,73]. Control of flavin movement to the *out* position has been linked to ionization of substrate hydroxyl [74], however, the definitive structural basis of this is not yet clear [75–77]. In the *out* position the flavin is reduced and the positive electrostatic environment of the active site induces the now dihydroflavin to return to the sheltered solvent-free *in* position from which NAD(P)^+^ dissociates [78]. Dioxygen interacts with FADH_2_ to form a *C*(4a)-hydroperoxyflavin that is stabilized to some extent by nearby residues. This intermediate acts as a weak electrophile, hydroxylating the nucleophilic target substrate [79]. Efficient hydroxylation is heavily dependent upon proximity of the target substrate and activation of the ring carbon most proximal to the hydroperoxy adduct [80,81]. The remaining *C*(4a)-hydroxyflavin eliminates water either prior to or concomitant with target product release [82].

Though clearly the paradigm, PHBH has a structural/mechanistic feature that makes it unique amongst Class A enzymes and to some extent undermines its value as a mechanistic example for the other class members. The key difference is that the movement of the flavin is linked to the substrate protonation state that is controlled by a proton shunt hydrogen bond network that connects the 4-hydroxyl of the target substrate deep in the active site to the solvent [77,83,84]. Evidence from the available structural data for other Class A enzymes appears to indicate a variety of control mechanisms for the dynamic behavior of the flavin and/or sections of peptide. This suggests that this ability to open and close the active site to solvent is a required part of the catalytic function and that it is tailored by both the structural requirements of the target substrate and the evolutionary lineage of each enzyme. For example, the enzymes PHHY, PgaE and CabE lack a hydrogen bond relay for activation of the substrate aromatic ring yet it is known that PHHY similarly regulates flavin movement to control reduction. Moreover, PHHY, PgaE, CabE, dihydroxypyridine hydroxylase (DHPH), and RebC possess apparently dynamic structural elements of the protein that act as a “lids” for the active site [7,73,85]. In another example, the flavin of *meta*-hydroxybenzoate hydroxylase (MHBH) adopts a predominantly *out* position prior to and after substrate binding and it has been proposed that only after reduction does the flavin move to the *in* position proximal to the substrate [86]. As stated, the Class A enzymes tend perform the same net reaction, the hydroxylation of an activated aromatic ring. The target substrate of which the ring is a part, however, can have considerable volume and shape differences in each enzyme system. This is consistent with available data that indicate principal differences between each enzyme relate to the target substrate recognition and the means by which this is linked to repeated dynamic movements that gate access to the active site.

### 2.1. Functional Assignment of Conserved Residues

**Figure 2:**
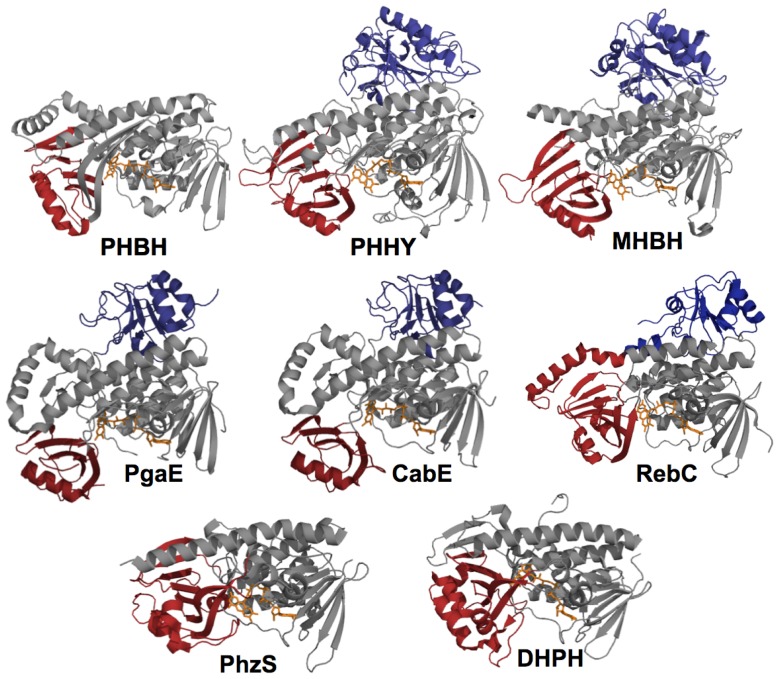
Comparison of Overall fold of Class A External Flavoprotein Monooxygenases. Domain I, shown in gray is typically known for FAD binding, Domain II in red lies at the interface of the active site and contributes to substrate binding, and Domain III in blue is thought to be involved in enzyme oligomerization. The structures shown are rendered from the following protein data bank files, 1PBE (PHBH), 1FOH (PHHY), 2DKH (MHBH), 2QA1 (PgaE), 2QA2 (CabE), 2ROC (RebC), 2RGJ (PhzS), 2VOU (DHPH).

**Figure 3:**
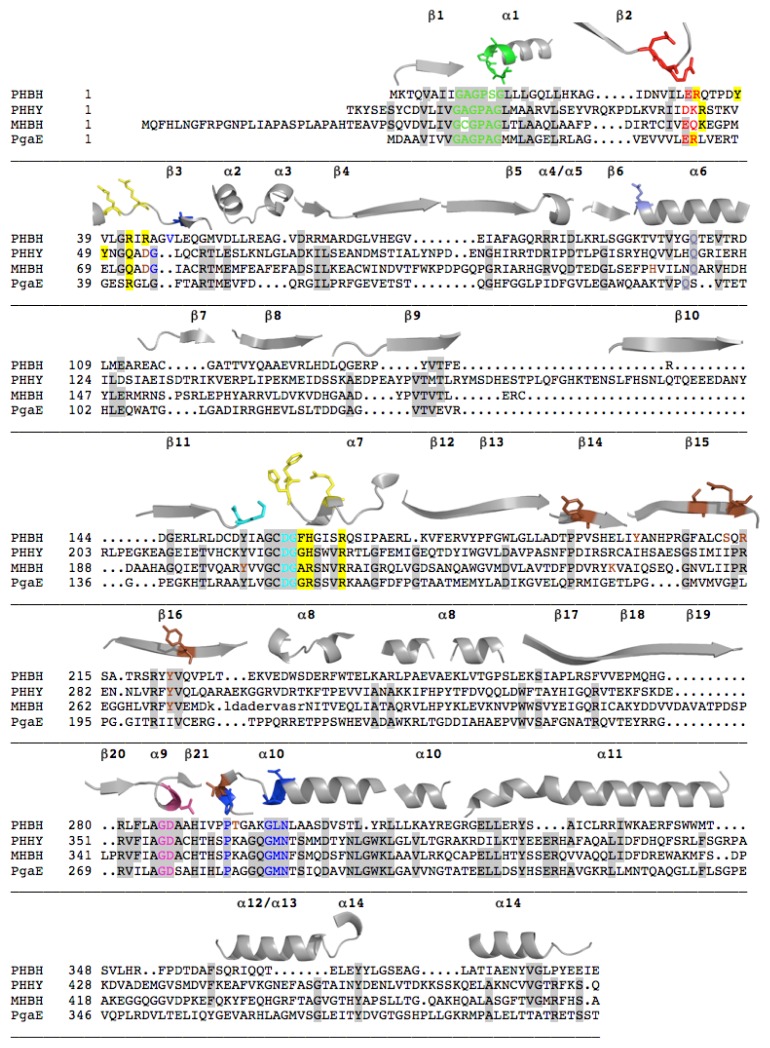
Sequence and Structural Alignment Comparisons of Four Class A FPMOs. Amino acids shaded gray or otherwise highlighted are either fully or partially conserved. The colored residues represent important motifs found in or near the enzyme’s active site. The 3-dimensional location of the amino acids is ostensibly the same for each color of residue. The secondary structure elements comprising the structure and the position of each positionally conserved residue. These were rendered individually from the structure of PHBH (PDB ID, 1PBE). A three dimensional representation of these colored residues is shown in Figure 4. The brown residues indicate substrate binding residues (the yellow or periwinkle residues are involved in flavin movement and/or NADPH binding) and the remaining colored residues are primarily FAD binding motifs. Note that only domains I and II are aligned. The complete sequences for PHHY, PgaE and MHBH include domain III (Figure 2). Accession numbers for the sequences are, PHBH (AAA88455.1), PHHY (AAA34202.1), MHBH (BAF34928.1), PgaE (AAK57522.1). The structures used for the 3D structural alignment were from protein data bank files, 1PBE (PHBH), 1FOH (PHHY), 2DKH (MHBH), 2QA1 (PgaE).

Free flavin is not capable of conducting well-orchestrated two, three and four-substrate catalytic reactions. As such the function of the flavin cannot be considered in the absence of the context of local protein environment. Figure 2 depicts the fold of the eight structurally characterized Class A enzymes. Each of the enzymes has two structural domains in common, the first, domain I (gray) is the large FAD binding region and the second, domain II (red) is a small *N*-terminal domain forming one edge of the substrate binding pocket. The active site is located at the interface of these two domains and consists of FAD and NADPH binding residues as well as target substrate binding residues. PHHY, MHBH, Pgae, CabE, and RebC extend further to form the small *C*-terminal domain III (blue) whose function is not known, but may participate in oligomerization in some instances. For example, PHHY is a homotetramer while MHBH, PHBH, and DHPH each form homodimers [73,85–87]. For PgaE and CabE, domain II is slightly rotated with respect to domain I and this necessarily increases the size of the substrate-binding pocket (Scheme 1) [7]. In these two enzymes, it is domain II that cooperates in dimer contacts, similar to PHBH, rather than domain III [7]. As such, the conserved function of domain III for PgaE and CabE is unclear. All three domains of PHHY, MHBH, PgaE, CabE, and RebC can be superimposed to some degree (Figure 2). The domains that contact the FAD in all five enzymes (I, II) contain a three-layer (ββα) sandwich where a three stranded antiparellel β-sheet (β8, β9, β10), a three-stranded parallel β-sheet (β1, β2, β10) plus a Rossmann fold form a β-sandwich with an overlaying helical section. [73,85]. In PgaE and CabE the primarily parallel β-sheet is mixed with anti-parallel β1, β2, β11, β19, and β20 elements [7] (Figures 2 and 3).

Figure 3 illustrates a sequence alignment of four members of the Class A enzymes that is colored to show the positionally conserved residues identified from three dimensional structure based alignment of PHBH, PHHY, MHBH, and PgaE. This alignment indicates the proteins share 22%–36% sequence identity (calculated using STRAP software) and is a representative sampling of the eight structurally characterized Class A enzymes that collectively show 15%–38% sequence identity. The occasionally low sequence homology belies the fact that the global fold of these proteins remains relatively constant. The sequence and structures of PHBH and PHHY have been compared and 77% of the residues can be superimposed three-dimensionally despite the fact that sequence identity is only 20% for structurally aligned residues [73,88]. PgaE and CabE are unique in that they have a sequence identity of ~70% [7]. In general for the Class A enzymes, the residues that are conserved are involved in FAD and NADPH binding and for the target substrates of similar structure, residues that participate in substrate binding will often also show homology.

**Figure 4:**
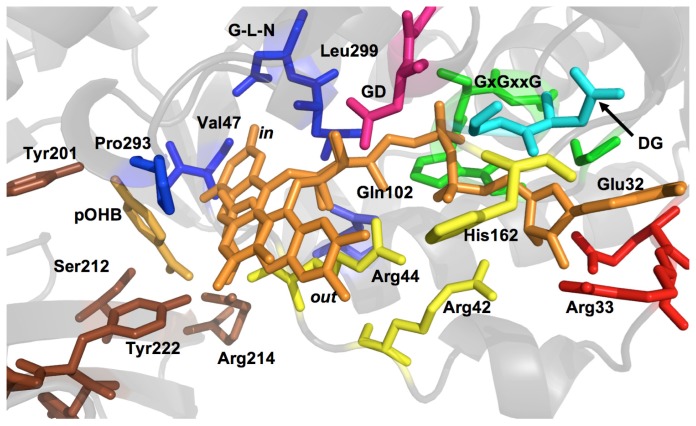
The active site residues of *p*-hydroxybenzoate hydroxylase (PHBH). Most residues depicted are conserved among the Class A enzymes discussed herein (PHHY, MHBH, PgaE, and CabE). The color scheme of positionally conserved residues will remain the same throughout all figures. The orange FAD cofactor is shown in both the *in* and *out* positions, the light-orange substrate *p*-hydroxybenzoate (pOHB) is in the target substrate binding pocket. The green, cyan, and pink residues correspond to three FAD binding motifs: *N*-terminal tail GxGxxG, DG, and GD, respectively. The red and neighboring yellow residues also take part in securing the flavin to the enzyme. The yellow residues are proposed to be critical residues for NADPH binding and stabilization of the flavin *out* conformation. Several additional yellow residues are implicated in NADPH binding but were omitted for clarity (see text below). The blue residues make important interactions with the isoalloxazine ring in the *in* position and/or couple to substrate binding. Most of these residues are found in the *re*-face polypeptide segment above the flavin. The conserved periwinkle residue (Gln 102) on the *si*-face of the flavin has been shown to act as a “latch” residue in PHHY to secure the flavin *out*, and makes similar contacts in the other enzymes. The brown residues help bind the substrate in the active site and the majority are specific to PHBH. With the exception of the FAD binding motif residues, most of the residues depicted are required to shift during catalysis.

Although the majority of the conserved residues play predictable or equivalent roles in each enzyme, some residues support other catalytic functions that are unique to the function of the enzyme in which they reside. Moreover, amino acids that may not align in a primary sequence comparison will often show alignment in three-dimensional space when structures are compared. In Figure 3 the functionally assigned residues of the active site are colored according to their depiction in Figure 4 that shows the constellation of important active site residues in PHBH. In the structural description that follows, unless stated otherwise, conserved residues will be numbered and colored according to their assigned function in PHBH from *Pseudomonas aeruginosa*.

In regard to overall fold, Class A FPMOs reside in the GR_2_-glutathione reductase subfamily, however, it is only regions that are involved with flavin binding that show significant sequence identity or similarity to this subfamily. A total of three FAD binding motifs are present in Class A enzymes that recognize and orient the adenosine, pyrophosphate, and ribityl moieties of FAD. The adenosine portion hydrogen-bonds to the protein primarily indirectly via intervening water molecules. Three hydrogen-bonding interactions with this group are observed in PHBH and only one is to the enzyme directly. Similarly, the pyrophosphate moiety makes as many as five indirect hydrogen-bonds (through waters) and three direct contacts. Amino acid residues that interact with the pyrophosphate group form the most conserved of the FAD binding motifs. It is the interactions with the adenine and pyrophosphate that provide the anchor for the cofactor as these groups are thought to undergo little, if any, movement throughout catalysis. In contrast, the isoalloxazine ring and part of the ribityl are in motion throughout the catalytic cycle and have almost exclusively direct hydrogen-bonding interactions, though this constellation of interacting residues is not at all well conserved [87].

There are many conserved Gly residues scattered throughout the primary structure, particularly clustered around or in conserved motifs. The pyrophosphate group motif, G*x*G*xx*G(*x*)_16–19_D(E) (PHBH 9–27, rendered in green in Figures 3 and 4) is also known as the βαβ-fold and starts in a loop region that is found between the *N*-terminal β-strand and the first α-helix. A tight turn of the main chain, adjacent to the first glycine residue, allows for the second glycine to orient itself in close proximity to the phosphates. In doing so, the positive dipole of the *N*-terminal end of helix α1 anchors towards the phosphate moiety, particularly towards the O_P1_ or O_P2_, presumably providing some amount of charge compensation. The final glycine in this sequence encourages tight packing of the terminal α-helix to an adjacent β-strand [21].

An aspartate-glycine pair at positions 159 and 160 (cyan in Figures 3 and 4) has proved to be both conserved and involved in FAD and NAD(P)H pyrophosphate binding motif. This sequence is located at β-strand 11 where the Asp and Gly residues tightly pack against and form hydrogen-bonds with residues ~163–165. The glycine residue enables this strand to turn sharply away from the FAD, anchoring the main chain in close proximity to the pyrophosphate group. This arrangement allows nearby histidine 162 (yellow in Figures 3 and 4, Arg in MHBH and PgaE) to be positioned in the putative NAD(P)H binding cleft [89] along with Arg42 (also yellow in Figures 3 and 4). The function of Arg42 had originally been only attributed to FAD binding as it forms a clear hydrogen bond to O3′ of the adenosine-ribose of FAD when the aromatic substrate is bound [90]. PHBH mutations in this position have been shown to primarily impact NADPH recognition by the enzyme [72,91]. However, this Arg residue is not conserved in PgaE or CabE and positionally it is substituted with a glutamate [7].

**Figure 5:**
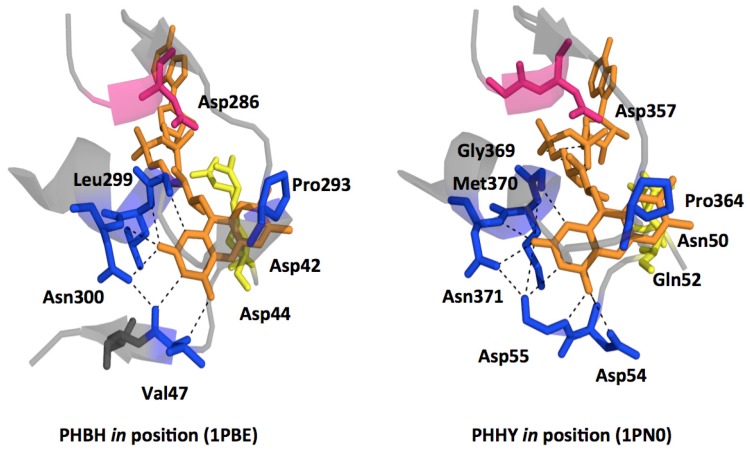
Flavin Interactions with the Flavin *re*-face and *si*-face polypeptide Observed in the *in* Position for PHBH and PHHY. The *si*-face (under flavin) and *re*-face (over flavin) polypeptide segments of PHBH (1PBE) and PHHY (1PN0) make polar contacts (black dashed lines) with the flavin isoalloxazine ring. All contacts made are within 3 Angstroms distance except for the bond lengths noted. Both sections of polypeptide work cooperatively to secure the flavin position. Hydrogen-bonding interactions are made between the *re*-face polypeptide and N(1) and O(2) sites on the isoalloxazine ring in both enzymes. The *si*-face polypeptide makes hydrogen-bonds to the N(3) and O(4) sites on the flavin ring system. A contact with a substrate liganding residue, Tyr289, to N5 of flavin is not shown.

A third conserved motif characteristic of the FAD binding domain is the FxxGD sequence from 282 to 286 (pink in Figures 3 and 4) where the conserved aspartate residue hydrogen bonds to O3′ of the uncyclized ribityl and appears to retain this interaction during catalytic conformational changes [73]. This sequence is along the *re*-face of the isoalloxazine ring in a cleft leading into the active site. There are also a number of conserved residues in the sequence after this motif that form a loop that passes through the active site such that proline 293 (blue, Figures 3 and 4) that is positioned in close proximity to the substrate. The position of Pro293 is thought to be dependent on the electrostatic environment surrounding the substrate, and is found in loop preceding an α-helix that contains the sequence GM(L)N (blue in Figures 3 and 4) that contacts the isoalloxazine ring of the flavin. In particular, the residues at the end of the α-helix, (Leu299 and Asn300 in PHBH) orient themselves close to N(1) and O(2) of the flavin isoalloxazine ring to form a network of four hydrogen-bonds (Figure 5) [73]. The apparent role of these interactions is to orient the isoalloxazine ring in the *in* position. This segment of the polypeptide therefore provides a direct link between a non-mobile interaction at the O3′ ribityl moiety, possibly defining the pivot point, and to the proline that moves with the development of charge at the substrate and thereby transmits this information to residues that interact with the isoalloxazine (see below).

This *re*-face polypeptide segment is likely a key component of the machinery by which substrate recognition (*i.e.*, binding and activation) is coupled to flavin position and hence reduction [84].

**Figure 6:**
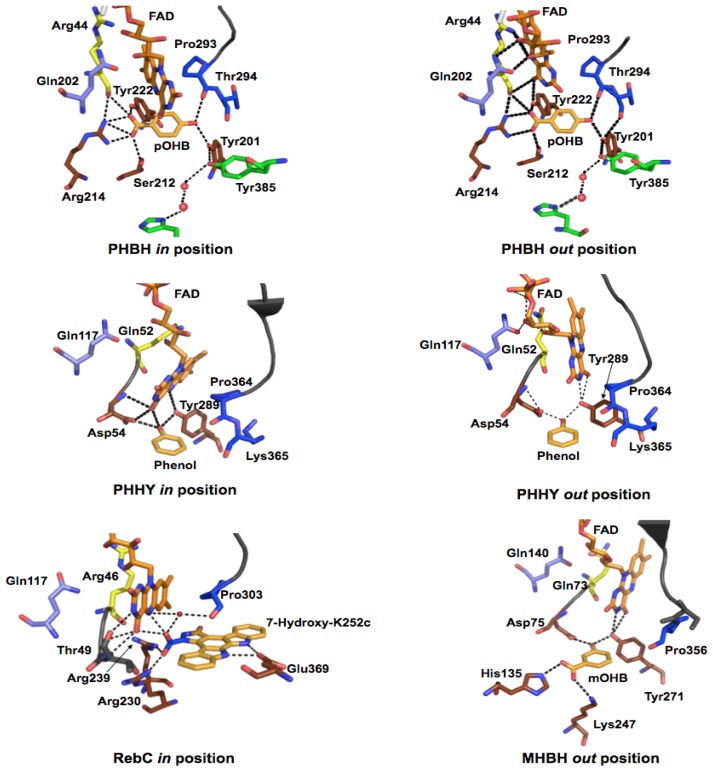
Representative target substrate binding sites and isoalloxazine ring positions for the class A external flavoprotein monooxygenases. Left—The substrate-binding pocket for PHBH, PHHY, RebC with the flavin *in* conformation is illustrated. In PHBH, the main chain carbonyls of Arg44 of the *si*-loop and Pro293 of the *re*-loop hydrogen-bond to each end of the aromatic substrate, pOHB. Other contacts are made with Tyr222, Arg214. Ser212, and Tyr201 to secure the target substrate. Residue Tyr201 links the substrate’s hydroxyl group in the solvent-free environment with His72 at the solvent interface by way of Tyr385 and two water molecules creating a hydrogen-bond network required for efficient catalysis. In PHHY, Asp54 of the *si*-loop and Tyr289 (which corresponds to Tyr222 in PHBH) make hydrogen-bond contacts with phenol as well as multiple contacts to the O(4) and N(5) of the isoalloxazine ring. No direct hydrogen-bond contacts are made between the substrate and the *re*-loop, only indirect contacts are made through the flavin isoalloxazine ring; Right—The substrate-binding pockets for PHBH, PHHY, and MHBH when the flavin establishes the *out* position. All hydrogen bonds remain the same in PHBH and PHHY except the bond from Tyr222/289 to the flavin which is reconstituted to the O(4) site in PHBH and the N(3) and O(4) site in PHHY. An additional hydrogen-bond in PHBH is made from flavin O(4) to Arg44 and from the *re*-strand Thr294 that lies within 3 Å of the start the hydrogen-bond network, Tyr201.

A second section of polypetide passes through the active site on the isoalloxazine *si*-face [67]. This section passes through the active site adjacent to both the flavin cofactor and the substrate-binding pocket. With flavin positional changes, the O2′ of the flavin ribityl experiences small movements (Figure 6) where hydrogen-bonds are formed between two nearby residues in each flavin position. These residues are staggered in a primary structure alignment of Class A enzymes but are positionally equivalent in three dimensional space. The first of these two residues of the *si*-strand is Arg44 (yellow in Figures 3 and 4, Gln52 PHHY and Arg42 PgaE) that seems to restrain the isoalloxazine ring position (Figures 4 and 5). For example, in PHHY the *si*-face polypeptide is observed to move in concert with the two known flavin conformations observed with this enzyme. Glutamine 52 in this case has been shown to act as a “hinge” that hydrogen bonds to the O2′, of the ribityl chain when the flavin is *in* and when the ring system moves to the *out* position this interaction is lost (Figure 6) [73]. In PHBH it has been shown that the arginine in this position (Arg42) is also involved in binding of NADPH [92]. In PHHY, for the *out* conformation a hydrogen bond is reconstituted to the O2′ oxygen from a Gln117 (shown positionally as periwinkle in Figures 3 and 4) acting as a “latch”. This latch residue is conserved in five Class A enzymes [73]. In MHBH, however, the residue in this position is too distant (3.85 Å) to establish a hydrogen bond to the O2′ ribityl (Gln140) [86] (Figure 6). Moreover, in the absence of structure for MHBH with the flavin in the *in* position, it can not yet be established if the conserved “hinge” residue (Gln73) forms the expected hydrogen-bond to the ribityl O2′.

Distant from the “hinge” in PHHY is Asp54 (MHBH Asp75) (Figure 6) that takes part in hydrogen bonding to the target substrate. This aspartate residue would appear to be important as it is one of two residues that help orient the substrate in proximity to the C4a position of the flavin (the site of dioxygen reactivity). In addition, the main chain of residues adjacent to the ‘hinge’ residue (Gly55 in PHHY and Val 47 in PHBH, shown in blue in Figures 3 and 4) form two apparent hydrogen-bonding interactions with N(3) and/or O(4) of the isoalloxazine ring that secure flavin in the *in* position (Figure 5). For PHHY, this strand structurally links substrate binding and flavin position. When the flavin moves to the *out* position, hydrogen-bonds with the isoalloxazine ring from these residues are lost and only those to the substrate remain (Figures 5 and 6). The *si-*face peptide segment is therefore implicated in many pivotal parts of the reaction mechanism, responsible for NADPH recruitment, hydrogen-bonding to the target substrate, defining the “hinge” point on the flavin ribityl group, and providing hydrogen-bonds to anchor the N(3) and O(4) of the isoalloxazine ring. Moreover, a connection exists between those interactions offered by the *re*-face peptide segment and those of the *si*-face as both are linked via hydrogen-bonding from Asn300 (*re*-face) and Val47 (*si*-face) (Figure 5).

For RebC only the flavin *in* position with target substrate bound has been observed while only the flavin *out* conformation has been crystallized for MHBH. For the latter, the target substrate binding contacts and substrate orientation with respect to the flavin is very similar to PHHY. The *si*-strand Asp75 and Tyr271 make substrate interactions and Tyr271 also anchor the flavin *out* conformation. The carbonyl group of the target substrate (mOHB) also hydrogen-bonds to residues His135 and Lys247. No substrate makes contact to the flavin when in the *out* conformation.

Not surprisingly, the residues that form the substrate-binding pocket differ for each hydroxylase and only a handful of residues are conserved for all Class A enzymes. The majority of the substrate binding residues are within the region spanning residues 201–225 (PHBH). However, the orientation adopted by 4-hydroxybenzoate in PHBH is distinctly different from those of native phenolic substrates observed in PHHY or MHBH (Figure 6). Residues that anchor 4-hydroxybenzoate in PHBH form hydrogen-bonds from the *re*- and *si*-side of the flavin ring, and the longitudinal axis of the substrate is roughly perpendicular to the isoalloxazine and toward the pyrimidine portion of the flavin (Figure 6) [87]. As might be predicted, this orientation exposes the *ortho* position of the substrate to the C4a position of the isoalloxazine. The substrates of PHHY and MHBH are also hydroxylated at the *ortho* position with respect to the activating substituent yet orient differently with the longitudinal axis somewhat aligned with that of the isoalloxazine such that the hydroxyl substituent points toward the dimethylbenzene portion of the flavin (Figure 6) [93]. As a consequence, the substrate binding residues are also in sufficient proximity and in appropriate orientation to form hydrogen-bonds with the isoalloxazine ring while in the *in* position.

The only partially conserved amino acid among the substrate binding residues is a Tyr (222 PHBH, 289 PHHY, 271 MHBH) that interacts with the respective carboxyl group in 4-hydroxybenzoate in PHBH, hydroxyl group of phenol in PHHY and hydroxyl group of 3-hydroxybenzoate in MHBH (Figure 6). In PHBH, five additional residue contacts to pOHB are observed, three of which orient the carboxylic acid and two others hydrogen-bond to the hydroxyl group. The main-chain of Pro293 of the *si*-face polypeptide segment, and the hydroxyl of Tyr201 interact with the 4-hydroxyl group; these interactions have proven to be vital in the catalysis of the enzyme [80,94]. The proximity of Pro293 serves as a means for the enzyme to sense the presence of the deprotonated target substrate by electrostatic repulsion (see below). Tyr201, is the first participant in the hydrogen-bond chain that dramatically lowers the pKa of the target substrate 4-hydroxyl (Figure 6) and acts cooperatively with the carbonyl of Pro293 to induce the repulsion that instigates flavin movement (Figure 6) [72,80,94].

Due to the single hydroxyl substituent, the target substrate in phenol hydroxylase can make only two hydrogen-bonding interactions with the active site. Both residues are situated close to the flavin and neither participate in a more extensive hydrogen-bond arrangements such as is seen with PHBH. However, these residues are essential in creating an appropriate environment to support target substrate hydroxylation [93]. One residue, Asp54, is part of the *si*-face segment that extends through the active site (Figure 5). The position of the second residue, Tyr289, (corresponding to Tyr222 in PHBH) is such that it is within hydrogen-bonding distance to both the phenol hydroxyl and N(5) of the isoalloxazine ring. In the *out* position this tyrosine residue continues contact to the substrate and forms a new interaction with the isoalloxazine ring N(3) and O(4′) (Figure 6). It has therefore been proposed that Tyr289 functions to aid the shift to the *out* conformation and anchors the flavin once it is *out*[93].

In addition to substrate binding residues, PHHY also possess four residues that stack with the phenol: Ile279, Met277, Ala366 and Gly367; the latter two residues are on the *re*-side segment (not shown) [73]. MHBH retains the same substrate hydrogen-bonding residues as PHHY, has a similar set of hydrophobic stacking residues (Ile260 and Leu258) but has added residues His135 and Lys247 that form salt-bridge anchors to the carboxylic acid of the target substrate (Figure 6) [86]. For MHBH, the *re*-strand proline is also conserved, however, it is not established if this residue engages in similar electrostatic based hinge effects as those proposed for PHBH.

Key structural differences of the Class A enzymes arise from differences in the target substrate structure. These differences influence the means by which the target substrate initiates flavin movement and how the target substrate gains entry to the active site. In PHBH, it has been proposed that the substrate gains access to its binding site from a widened solvent channel that is present on the *re*-side of the isoalloxazine ring when the flavin is in an intermediate position denoted as the *open* conformation [67,72]. In PHHY, two conformational changes occur in order for substrate binding to take place. While access to the active site relies on flavin movement to an *out/open* position, it is also contingent on additional large conformational change of two loops, bounded by residues 170–210 and 43–52. These loop regions are thought to form a lid that closes off the active site from solvent [73]. A similar “lid” of partially ordered helix in RebC becomes ordered and caps the active site entrance once the substrate has associated. Since the substrate of RebC is too large to gain access via an *open* flavin position, it is thought that the lid mechanism is an adaptation to permit entry [6]. PgaE and CabE contain a partially mobile loop formed by residues 221–226 that are thought to behave similarly [7]. For DHPH it has been proposed that displacement of a loop region in addition to the flavin *out* conformation is necessary for substrate to enter the active site alongside the *si*-face polypeptide segment [85]. Peptide displacements in place of or in addition to isoalloxazine movement must occur in a coordinated manner if their function is to close off the active site and shelter the catalytic steps from solvent at specific times during catalysis.

Class A enzymes may also employ tunnels to aid target substrate access to the active site. There are two very well defined channels in MHBH, one solvent-free channel containing predominantly hydrophobic residues and the other rich in polar residues containing many water molecules. It was proposed that the solvent-free channel whose opening is lined with positive residues is for substrate binding and entrance to the active site, while the second channel has characteristics for product release [86]. In addition, PgaE, CabE, PhzS and RebC also contain tunnels similar to that observed in MHBH [95]. In all cases, the tunnels span from the surface of the enzyme to the active site, ending directly below the C(4a) position of the flavin [6,7]. For PgE, CabE and certainly RebC it is not clear that it is feasible for the bulky target substrates of these enzymes to access the active site through these channels and these may instead be a vestige of the progenitors of these enzymes. It is of value to note that while tunnels have been consistently observed in the structures of the Class A flavoprotein monooxygenases, the function of these structures has not yet been rigorously investigated.

### 2.2. The Mechanism of the Class A Fpmos

Almost 50 years of continuous research has been directed toward the elucidation of the catalytic mechanism of *p*-hydroxybenzoate hydroxylase (PHBH). As such, PHBH is a candidate for having the most thoroughly understood mechanism of any multi-substrate enzyme [58]. As is clearly evident from the structural description above, PHBH is a somewhat unique member of the Class A flavoprotein hydroxylases (Figure 6). Despite this, it has been adopted as the prototypical standard for the development of mechanistic ideas for the other members of the FPMOs and in some instances other mechanistically related flavoproteins. The amenability of this enzyme to pre-steady-state rapid-mixing methodologies and the ability to study the reductive and oxidative half reactions entirely independently has provided a wealth of clear and largely un-refuted mechanistic data. Site-directed mutagenesis in parallel with crystallographic studies has played a pivotal role in shaping the understanding of how catalytic residues cooperate to cause the observed mechanistic steps. For no other flavoprotein hydroxylases is the mechanistic picture as complete. As such, in the following section we will describe the steps of a single catalytic cycle of a Class A flavoprotein aromatic hydroxylase chronologically drawing most often from the literature available for PHBH.

Class A external flavoprotein monooxygenases use a single flavin cofactor and a single peptide to orchestrate reactions involving three substrates: a target substrate that is typically aromatic, NADPH, and O_2_. Each of these substrates has very specific and very different requirements for acquisition and reactivity. The kinetic mechanism of these hydroxylases indicates a preferred order of substrate association. This order does not correlate with the order in which the substrates are consumed by the enzyme, as the target substrate is preferentially bound before all other substrates but is not altered chemically until the penultimate catalytic step after the acquisition of two other substrates and the departure of one product. The enzyme accomplishes this by a series of flavin conformational states that effectively couple target substrate binding to hydroxylation by sensing proton abstraction from the target substrate. The purpose of these states is to avoid squandering the two electrons brought to the reaction by NADPH in the form of futile dioxygen reduction to hydrogen peroxide [96].

**Scheme 3:**
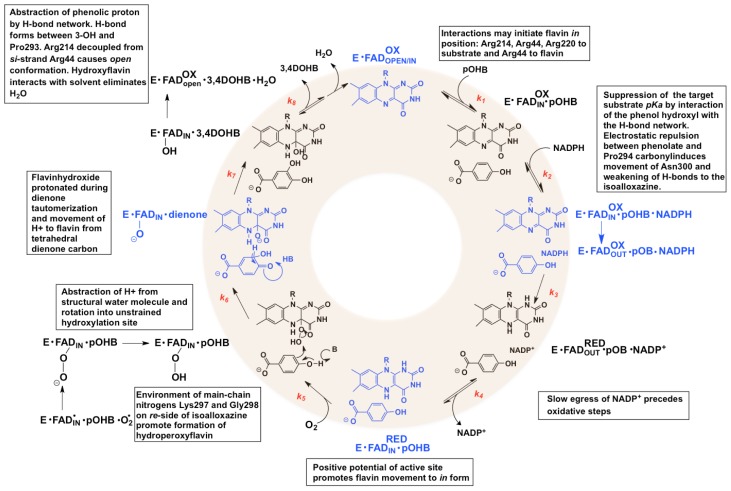
The catalytic cycle of *para*-hydroxybenzoate hydroxylase. Each of the states shown has been observed either with the wild type enzyme, in the presence of analogs of the target substrate or with mutant forms of the enzyme. The bounds of the circle represent the boundary of the enzyme, schematically depicting where in the cycle that ligands add to or depart from the enzyme.

All tractable FPMOs can be studied independently at three stages of catalysis, target substrate binding, the reductive half reaction, and the oxidative half reaction. For the Class A FPMOs addition of the aromatic substrate greatly stimulates the rate of reaction with NADPH [41]. NADPH association in turn induces the flavin to the *out* position where two electrons from the nicotinamide ring are transferred to it as a hydride [76,84]. The anionic charge brought to the flavin by reduction causes the isoalloxazine to retreat to the largely positive potential, solvent-free active site environment within the enzyme [97,98]. NADP^+^ then dissociates leaving the reduced flavin available to reduce molecular oxygen and form the key *C*(4a)-hydroperoxyflavin intermediate that is stabilized in part by the absence of water. The substrate undergoes electrophilic attack by this flavin intermediate and the distal oxygen of the peroxo group is delivered to form an non-aromatic hydroxylated intermediate and a *C*(4a)-hydroxyflavin [99]. This flavin species then decays to form water and the oxidized flavin. The decay of the hydroxyflavin is either coincident with or occurs prior to the dissocation of the hydroxylated product [82]. The reaction mechanism and structural changes associated with each catalytic step for PHBH are detailed in Scheme 3. The following sections will elaborate on what is known for each stage of catalysis.

#### 2.2.1. Substrate Binding

The understanding of substrate binding in the Class A monooxygenases is incomplete. However, it is clear that there is no single strategy that is employed by all enzymes and each ligand binding mechanism is tailored to the target substrate that is bound. The major differences between enzymes within Class A tend to be with regard to the orientation of target substrate binding (Figure 6), the target substrate pocket volume, and possible differences in the route by which the substrate gains entry to the active site. These differences are apparent in the residues that line the entrance of the active site and in the residues that secure the substrate in the binding pocket.

Enzyme structures and site-directed mutagenesis experiments have become the avenue to understanding the mechanism of substrate binding in PHBH. Only recently have high-resolution, substrate bound structures that capture multiple flavin conformations been observed. Initially, both *in* and *out* conformations were captured by these structures that did not seem to provide a means for substrate to access the active site. A crystal structure was then solved with the analog 2,4-dihydroxybenzoate that showed a new position for the flavin that emphasized the apparent importance of Arg220 whose side chain had moved to a different conformation where it formed hydrogen-bonds to Arg44 on the *si*-face segment and the target substrate (not shown) [66,70]. The structure of the Arg220Gln form of PHBH crystallized in complex with NADPH also captured an *open* flavin position that clears a solvent path to the interior of the protein [72]. This channel is now thought to be the means of access for target substrate and a departure route for the product. Not only does the *open* position allow for substrate binding, but it also prevents the flavin *re*-side from contacting the NADPH nicotinamide prior to substrate association, suggesting that this conformation helps to regulate the order of addition and may account for why the rate of reduction is slower by five orders of magnitude in the absence of the target substrate [41].

It is important to note that the substrate-free enzyme does not remain in a static *open* position, and it has been determined that the flavin would be in rapid equilibrium between the *open*/*in* conformations with a possible preference the *open* state [70]. Raman spectroscopy has also been able to show that the O4′ flavin group of substrate-free enzyme is in a dynamic hydrogen-bonding environment with water molecules, very different to the enzyme-target substrate complex that forms a largely static hydrogen-bonding and charge pairing interactions [100]. To study each of these states individually, mutants have been prepared that vary the volume of the residue at the Arg220 position and directly affect the flavin conformational balance. A glycine residue at this position stabilizes the *in* position and limits the rate of substrate binding and similar results were observed for both the Asn300Asp and Lys297Met mutants whose flavin *in* form was stabilized [101,102]. A valine in the 220 position stabilized the *open* state and restored rapid substrate binding [58,67] but the affinity of substrate for the binding pocket was considerably weaker. Presumably this is because target substrate association requires the *open* form for access and the *in* form to close the active site and form all required ligand interactions [68]. A fairly extensive array of hydrogen-bonds hold the substrate in its binding pocket, the substrate carboxyl is held in place by three hydrogen bonds from Tyr222, Ser212, and the main chain of Arg44, and a salt bridge with Arg214 (Figure 6). The substrate is further secured in place by hydrogen-bonds to the phenol hydroxyl from the main chain Pro293 carbonyl and the hydroxyl of Tyr201.

Substrate binding in PHBH has been proposed to involve a minimum of two steps, but an explanation for why the flavin retreats to the *in* position when the target substrate is bound has not been proposed. However, from the Arg220Gln structure it had been concluded that the *si*-face polypetide was rotated in the *open* position, decreasing strain in the main-chain [67]. This rotation was proposed to be linked to the displacement of Arg214 from the substrate-binding pocket so that it could no longer hydrogen-bond indirectly to Arg44 of the *si*-face polypeptide (Figure 5) [71]. The Ala45Gly structure confirmed this reasoning as this variant stabilized the flavin *in* position with pOHB bound and had Arg214 in the substrate pocket hydrogen-bonding to Arg44 by way of the substrate carboxylate group. The re-establishment of this interaction resulted in restoration of strain in the *si*-face polypeptide. These interactions between the target substrate and residues Arg214, Arg44, and Arg220 link flavin position with target substrate association.

The substrate-binding pocket of PHBH accommodates close packing of neighboring residues adjacent to the substrate that allow very little room for variation of ligand shape without impacting the orientation of the ligand and the efficiency of catalysis. Mutant forms of PHBH that alter the target substrate binding pocket often broaden the specificity and/or change regiospecificity of hydroxylation [76]. However, the shape and volume of pOHB is not unique and wild-type PHBH bases its substrate specificity on detection of an ionizable *para*-phenolic substrate while investing the majority of the binding energy in interactions with the carboxylate [76,84,103]. Residues Tyr201 and Tyr385 pack closely to the 4-hydroxyl and are the first participants in a chain of hydrogen-bonding residues and waters that extend from the target substrate hydroxyl to the surface of the protein. The mutants Tyr201Phe and Tyr385Phe have been shown to lose a degree of substrate specificity permitting accommodation of target substrate analogs with greater volume [80,81,104]. However, the reductive half reaction and hydroxylation efficiency of these mutants is dramatically negatively impacted by disruption of the hydrogen-bond relay [80].

PHHY shows narrow substrate specificity for most bacterial forms of the enzyme. The eukaryotic phenol hydroxylases, however, accept a broader range of substrates. The PHHY binding pocket does have small cavities around the aromatic ring that could accommodate other ring substituents though none are large enough to accommodate a carboxylic acid moiety and pOHB is not able to bind to PHHY and there is no substrate that is known to be common to both enzymes [73]. PHHY has the capacity to hydroxylate fluoro-, chloro-, amino-, and methyl-substituted phenols [105,106]. Similar to PHBH, the enzyme is not required to bind the phenol substrate for flavin to become reduced by NADPH, however, the affinity for NADPH and the rate constant for reduction increases with target substrate binding [107,108].

There has been debate as to the protonation state of the phenol substrate in PHHY when initially bound to the enzyme and throughout catalysis. The first proposal suggested that substrate bound the enzyme in phenolate form, due to increase in affinity observed at higher pH values [108,109]. However, binding experiments using *p*-nitrophenol showed the neutral form binding to the oxidized enzyme [110]. ^19^F-NMR confirmed this in that a fluorinated phenol also bound to the oxidized enzyme in neutral form but was deprotonated with reduction of the flavin. It was revealed that a pKa of ~6.5 was established on an amino acid in the active site with flavin reduction and this residue acted as a base to help deprotonate the substrate [111]. The apparent increased acidity of the target substrate in the active site of PHHY belies the fact that it does not possess a hydrogen-bonding relay as observed in PHBH.

The crystal structure of PHHY has provided structural insight as to how the enzyme binds the target substrate. As discussed earlier, PHHY is a homotetramer and two distinct conformations were observed in the structure of the oligomer. In the first, two short helices spanning residues 170–210 apparently undergo a conformational change that results in a shift to and from the entrance to the active site and appear to form an open and closed conformation. This “lid” has been suggested to regulate solvent and substrate accessibility to the active site [73]. However, as in PHBH, flavin movement *in* and *out* is also observed. The crystal structure showed that the loop closed and flavin *in* conformations corresponds to one set of protomers and the lid open and flavin *out* corresponds to another, but curiously, the target substrate occupies the active site of both forms. Assuming that the structures are representative of catalysis, this may suggest that the flavin *in* conformation is not the prodominent form for PHHY when substrate is bound, as it apparently is in PHBH-pOHB structures [87].

For RebC the substrate-free crystal structure has a flavin *out* conformation in contrast to an *open/in* flavin for PHBH. Due to the bulky substrate of RebC the characteristic *open* position would not allow enough space for its entrance into the active site. Comparison of the substrate-free and substrate bound structures also revealed a large structural change of a disordered helix that became ordered, coined the “melting helix”, that caps the active site with target substrate binding [6].

Similarly, MHBH resides in a predominantly *out* flavin conformation prior to substrate binding and 3-hydroxybenzoate is unable to enter the active site of MHBH via the flavin *re*-side due to a steric block provided by Tyr317 that makes a stacking interaction with the isoalloxazine [86]. It therefore appears that MHBH requires some other means for target substrate entrance to the active site that is not observed in either PHBH or PHHY. As stated above, tunnels oriented approximately 180° from the putative NADPH binding region lead to the active site in MHBH. These two paths, converge into one single tunnel that leads to the substrate binding pocket. It was proposed that the substrate initially binds to a His120 and Lys247 on the solvent interface (which is a similar residue configuration to that in the substrate-binding pocket) (Figure 6). The substrate can then diffuse through this primarily hydrophobic tunnel to the active site [86].

Only one substrate-free structure has been solved for PgaE and CabE, so the means by which substrate binding takes place is speculative. However, computer-modeling using a docking program for PgaE resulted in several modes by which the substrate could orient in the active site. One position has the substrate ring towards the NADPH binding pocket and orients the site of hydroxylation 4.2–4.5 Å from C(4a) of the flavin. Despite having a tunnel similar to MHBH, it was suggested that the target substrate enters instead through the active site with help from a conformational movement of loop residues 221–226, proximal to the putative NADPH binding site [7].

#### 2.2.2. Reductive Half Reaction

The reductive half reaction is a marvel of choreographed events that are each heavily dependent upon electrostatic environment of the active site. During this phase of catalysis the target substrate has been acquired and its binding is linked to a sensing mechanism that stimulates the flavin to change conformation with the association NADPH to a position that promotes hydride transfer [76,84]. As such the reduction process is dynamic and relies on several sequential conformational changes that certainly in PHBH and possibly PHHY, are linked directly to the protonation state of the target substrate. Stopped-flow techniques have enabled observation and measurement of the discrete catalytic steps before, during and after flavin reduction. These data added to similar studies using mutant enzyme forms and crystallography studies have allowed the assembly of a surprisingly detailed picture.

Target substrate binding is not a requirement for the flavin to be reduced by NADPH [41,82], however, when the target substrate binds it facilitates flavin movement to the *out* position with the binding of NADPH which exposes the *re*-side of the isoalloxazine ring system [66,69]. As such the binding of the target substrate increases the rate constant for reduction by ~10^5^-fold [41] and this dramatic enhancement is how the enzyme couples reduction to hydroxylation of the target substrate and avoids wasting reducing equivalents from NADPH when the substrate is absent. This strategy is only necessary in the Class A enzymes as the *C*(4a)-hydroperoxyflavin that is formed in these enzymes is not hyperstabilized and will rapidly decomposes to oxidized flavin and hydrogen peroxide if the target substrate is not bound. The Class A strategy for efficiency is thus different from that of most other classes of FPMOs that advance the reaction to the hydroperoxyflavin without the contingency of target substrate binding and consequently do not link substrate binding with flavin reduction [112].

**Figure 7:**
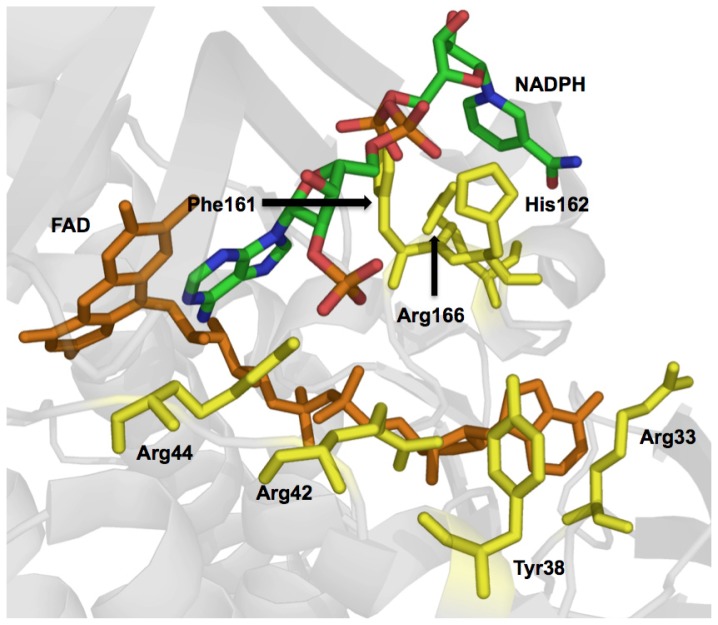
The Structure of PHBH Variant, Arg220Gln with a Molecule of NADPH Bound (PDB ID:1KOJ), and the Positions of Residues Implicated in NADPH Binding in this and Other Studies. In the structure the adenine of NADPH is positioned in a stacking orientation with the isoalloxazine ring and the nicotinamide ring is oriented above the binding cleft between Phe161 and His162. It is important to note that in this binding mode that only two direct hydrogen-bond contacts are made to NADPH. In the rationalization of the structure it had been proposed that when hydride transfer is made, NAPDH forms a hooked conformation to orient the nicotinamide in close proximity to the N(5) flavin site [72]. In other studies a range of different residues that roughly track the binding cleft of the FAD have been implicated by mutagenesis as contributors to both NADPH binding and NADPH/NADH specificity. These residues include Arg33, Tyr38, Arg42, Arg44, Phe161, and Arg166 [92,115,116].

In the PHBH structure the substrate sensing/activating hydrogen-bond chain has been shown to span a distance of 12 Angstroms from the substrate hydroxyl group, through Tyr201, Tyr385, two buried water molecules, and His72 at the solvent interface (Figure 6) [83,84,113]. It has been shown that the network senses the presence of substrate and promotes the movement of the flavin to the *out* position [114] where it can more readily contact the NADPH nicotinamide. Despite all that is known of the reductive half reaction, identification of the binding pocket for NADPH has remained elusive. The lack of a second Rossman fold in the Class A enzymes dictates that the binding site for NADPH will not be identified from a structural or sequence motif precedent. The only crystal structure of a Class A FPMO in complex with NADPH was solved for the Arg220Gln variant of PHBH [72]. This structure has the reductant bound approximately reversed from what might be intuitively predicted with the adenine base buried behind the *si* face of the flavin and the nicotinamide ring distant from the flavin isoalloxazine (Figure 7).

This binding orientation is at odds with the observation of flavin-to-nicotinamide charge-transfer bands that are observed during reduction and indicate close proximity of the two bases [82,41,117]. Mutagenesis studies have provided the bulk of the evidence for which residues interact with NADPH. These data suggest that NADPH associates with a set of aromatic and positively charged residues that line a binding pocket adjacent to the FAD binding cleft [91,92,115,116] (Figure 7). The observation most fundamentally at odds with the structural data is the observation that the key NADPH/NADH specificity determinants are provided by Arg33, Tyr38 and Arg42, strongly suggesting that NADPH binds in the opposite orientation in normal catalysis. Collectively these studies suggest that the FAD and NADPH molecules may adopt approximately side-by-side positions during reduction. Additional evidence for the close association of the nicotinamide and isoalloxazine rings is found in the egress of NADP^+^ that immediately follows reduction of the flavin. The dissociation of NADP^+^ is required to permit the reaction of the flavin with dioxygen, suggesting that the oxidized nicotinamde ring sterically blocks access to the flavin N(5) [41,118].

The hydrogen-bond network in PHBH is influenced by the protonation state of His72. When this distant residue is neutral protons shunt toward the solvent deprotonating pOHB. The phenolate that is formed repels the carbonyl of the nearby Pro293 residue [84] that then contributes to the movement of the *re*-face peptide backbone [94]. As described earlier, Pro293 is located on the *re*-face loop that establishes many hydrogen-bond interactions to the isoalloxazine in the *in* position as well as makes contacts to the *si*-face polypeptide via Asn300. The shift of those isoalloxazine binding resides along the *re*-strand, particularly Asn300, results in the disruption of hydrogen-bonds that retain the flavin in the *in* position (Figure 6) initiating the movement of the flavin from the *in* to the *out* conformation. The pivot that occurs at C(2) of the ribityl moves the isoalloxazine 7–8 Å to the solvent interface where it can contact the NADPH nicotinamide [84].

Individual variants of the residues for the first participants of the hydrogen-bonding network (Tyr201 and Tyr385 to phenylalanine) resulted in no significant change to the enzyme structure but caused respective 10- and 100-fold decreases in the rate constant for reduction [80]. Mutation of His72 to asparagine resulted in two populations of enzyme at neutral pH, one undergoing rapid reduction the other reducing very slowly. The proportions of each population was observed to be pH dependent and proposed to be a result of an enzyme fraction that formed the substrate phenolate and hence achieved the flavin *out* position and another fraction that remained phenolic and resided in the *in* conformation. From these variant enzymes it was concluded that the deprotonation of pOHB was required for rapid reduction [113]. Water molecules of the hydrogen-bond network are equally critical, however, only one of the two waters believed to participate (spanning the gap from Tyr385 to His72) has been observed crystallographically with the native substrate in the active site [74]. The second water molecule is observed, however, when *p*-aminobenzoate is bound [69]. It has been proposed that rotation of the hydroxyl groups of the target substrate, Tyr201, and Tyr385 would encourage the second water entry and would initiate polarization of the hydrogen bonds towards His72 [74].

In PHBH the electrostatic environment adjacent to the substrate 4-hydroxyl is predominantly positive. This environment is necessary to lower the substrate pKa and stabilize the phenolate form of target substrate [102]. A number of residues in the active site help establish both the strength and shape of a positive field that is adjacent to and influences the proton network. At least four positively charged residues contribute; Arg45 on the *si*-face polypeptide segment, Lys297 on the *re*-face segment along with Arg220 and Arg214 that both make hydrogen-bonding interactions with the substrate (Figure 6). A decrease in the positive electrostatic character caused by the mutation Asn300Asp decreased the rate constant of reduction by 330-fold, despite that this mutation would altered hydrogen-bonds to the O(2) of the isoalloxazine while in the *in* conformation and may have been expected to make the flavin more susceptible to reduction by promoting the *out* form. Instead the decrease in reduction rate constant was primarily attributed to an inability to suppress the pKa of the substrate [101]. A charge neutral mutation, Arg220Lys, did not disrupt target substrate deprotonation, however the NADPH binding affinity increased by 40-fold, and the reduction rate was slowed by one order of magnitude [75].

Hydride transfer results from the proximity of 4-R hydrogen of NADPH and the N(5) of the isoalloxazine ring [119]. After reduction, NADP^+^ dissociates and the newly acquired negative charge on the reduced flavin draws it back to the positive electrostatic field of the active site [102]. However, the active site is thought to only be able to support a single negative charge and therefore the substrate is concomitantly re-protonated by the hydrogen-bond network [113]. At this point, the target substrate and flavin lie in an environment where the reaction with molecular oxygen can occur free of the destabilizing influence of water.

The reductive half reaction is less well characterized for PHHY; however several residues within the active site have been shown to play an important part. Data would suggest, at least for selected substrates, that deprotonation of the aromatic substrate does not occur until after reduction [111]. Moreover, no clear evidence has shown what conformation the enzyme or the flavin reside in prior to reduction, however it is assumed an *out/open* positions of the flavin and “lid” peptide respectively would facilitate such chemistry [73]. As stated above, the substrate is held in the binding pocket by two just residues, Tyr289 and Asp54 that also form a network of hydrogen-bonds to the isoalloxazine ring when the flavin is in the *in* conformation (Figures 5 and 6). The Tyr289Phe PHHY variant increased the redox potential of the enzyme by 30 mV yet the reduction rate constant was slowed by 12-fold and it was suggested that the flavin *out* form was destabilized [93]. The mutant Pro364Ser on the *re*-face polypeptide (Figures 5 and 6), caused an increase in the reduction rate constant in the presence of phenol by disrupting the rigidity of this segment. It was argued that increased motion within the segment diminished the net strength of hydrogen-bonding interactions that position the flavin *in* resulting in a larger population of flavin *out*[120]. Although aiding reduction, this mutation proved to have detrimental effects upon the oxidative half reaction (see below). However, when the substrate resorcinol is bound to the Pro364Ser mutant a compensatory effect is observed. The rate constant for reduction returned to that observed with WT which suggested that the hydrogen-bonds made by this substrate to the *re*-face polypeptide residues help to reinforce the peptides position [120]. It is proposed, as it is for PHBH, that after hydride transfer the isoalloxazine ring moves to the sheltered *in* conformation to perform hydroxylation.

Compared to PHBH and PHHY, PgaE, CabE, RebC, PhzS, and DHPH are relatively new additions to the research record and many of the finer details of the reductive half reaction are not known for these enzymes. However, mutant studies for PgaE and CabE have identified important residues in the active site [7]. These enzymes do not seem to have a proton relay to activate the target substrate as is observed in PHBH, and no residues in the substrate-binding pocket are obvious candidates to act as base. For PgaE and CabE, Asn289 has been shown to form hydrogen-bonds to the O(2) of the isoalloxazine ring in the *open* conformation of the enzyme. Mutation of this residue to an aspartate eliminated the hydrogen-bonds to the flavin and added a local negative charge to the active site. The net effect of this mutation was almost complete inactivity of the enzyme (though the specific catalytic steps affected have not yet been established) [7].

#### 2.2.3. The Oxidative Half Reaction

In the presence of dioxygen, the oxidative half reaction commences with dissociation of the oxidized nicotinamide ring of NADP. A collision of the reduced flavin with molecular oxygen results in the transfer of a single electron between these species [28,121]. This first electron transfer from the reduced flavin to molecular oxygen is thermodynamically unfavorable and limits the rate constant for this reaction [71]. The transfer forms two radical species, the superoxide anion and the flavin semiquinone radical [122]. The second electron transfer from the flavin radical to superoxide requires spin inversion that is followed by facile radical recombination of the two species to form a *C*(4a)-peroxyflavin intermediate. It is assumed that both of these steps occur when the flavin resides in the *in* position, sequestered from solvent so that radical recombination and solvent induced elimination does not lead to the futile production of H_2_O_2_[66]. The stabilization of this flavin adduct is supported by both the largely positive electrostatic environment surrounding the flavin and the main-chain amides of Lys297 and Gly298 (PHBH) [79]. Modeling experiments suggest that the distal oxygen of the peroxyflavin anion may initially orient itself toward a water molecule in order to acquire a proton. The resulting *cis*-flavin *C*(4a)-hydroperoxide then rotates around the *C*(4a)–O bond towards the hydroxylation site and orients closely to the target substrate [79]. This *C*(4a)-hydroperoxyflavin intermediate is the first of three transient species that are commonly observed during the oxidative half reaction (Scheme 3) [28]. Repositioning of the distal oxygen of this species so that it is proximal to the target substrate is influenced by the volume of adjacent active site residues. The Ala296Val mutant caused the repositioning of the hydroperoxide and produced a 200-fold decrease in the rate constant of hydroxylation [123].

The second step of the hydroxylation reaction relies on electrophilic attack of the flavin hydroperoxide on an activated position of the ring of the target substrate. This reaction yields a flavin-*C*(4a)-oxide anion and a non-aromatic form of the product. As might be anticipated, the link between the substrate and the solvent in PHBH make electrophilic attack by the flavin hydroperoxide a pH dependent process [124]. When the hydrogen-bond network is disrupted, such as is the case for Tyr201Phe, hydroxylation is slowed by 10^3^-fold [32,80,125]. Consistent with this, the target substrate analog, *p*-aminobenzoate (pAB), binds to PHBH with similar affinity to pOHB, does not promote reduction yet is hydroxylated normally and this reaction is not influenced by pH changes [28,126]. Quantum mechanical calculations indicate deprotonation of the target substrate lowers the energy barrier and increases the hydroxylation rate constant by as much as 4 × 10^3^-fold [127]. It has been suggested that hydroxylation actually commences with a fully protonated substrate [83] and that the nucleophilicity is only enhanced by deprotonation in the hydroxylation transition state [125,128].

The electrostatic environment of the active site supports and hones the catalytic chemistry of all enzymes. For PHBH the overall fold is thought to have a dramatic effect by increasing the rate constant of hydroxylation by upwards of 10^2^–10^3^-fold [71]. The pKa of the 4-hydroxyl of pOHB is ~7.1 when bound to PHBH, at least three pH units below that of free pOHB [80,129]. In the Asn300Asp and Lys297Met mutants of PHBH that each decrease the positive charge in the active site by one, a 50-fold decrease in the rate constant for hydroxylation was observed and attributed in part to an inability to sufficiently lower the pKa of the substrate even though both kept the substrate’s hydrogen-bond network intact [101,102]. Similarly, an increase in the positive electrostatic environment in Glu49Gln mutant resulted in an order of magnitude increase on the rate constant for hydroxylation [78].

The pKa of the leaving distal peroxy oxygen is equally important in controlling the rate of hydroxylation as shown by modification of the leaving group by adding electron-withdrawing substituents to the 8-position of the flavin. These substituents lower the adduct pKa and each result in more rapid hydroxylation rate constants [129,130]. In addition, it is thought that the flavin O–O bond lengthens with the increase in lowest unoccupied molecular orbital occupancy on the protonated distal oxygen that in turn initiates C–O bond formation on the target substrate. Furthermore, stabilization of the proton on the leaving oxygen atom by the partial negative charge on the carbonyl backbone of Pro293 (~2.0 Å away in the transition state complex) may also aid hydroxylation [127].

Two species are formed with target substrate hydroxylation. The first is the flavin hydroxide that retains the proximal oxygen atom of what was the hydroperoxo moiety and the second is a hydroxylated, non-aromatic ring species that is *sp3* hybridized at the site of hydroxylation (Scheme 3). In PHBH, this is a non-aromatic dienone species that is short-lived and not observed to accumulate with the native substrate due to rapid re-aromatization with reprotonation of the 4-OH of the target substrate (Scheme 3). Evidence for the existence of this species has come from substrate analogs that have additional activating substituents *meta* to the native target substrate’s activating substituent. The non-aromatic hydroxylation products of these analogs are stabilized by resonance and accumulate in the reaction and can be shown to decay to yield the expected target product [76,99]. The *C*(4a)-hydroxyflavin is formed by the proton transfer from the tetrahedral carbon that formed on the target substrate during hydroxylation to the flavin hydroxide during re-aromatization [125] (Scheme 3).

The rate constant for hydroxylation for PHHY is slower than for PHBH and it has been assumed that this is a result of the absence of the more elaborate hydrogen-bond relay that PHBH has to activate the target substrate [110]. Analogous to what is observed in PHBH, after PHHY undergoes hydride transfer the flavin pivots into the active site and the “lid” residues close off the entrance to the solvent. The *C*(4a)-hydroperoxyflavin is formed and residues Asp54 and Tyr289 hold the substrate in close proximity to the flavin where the electron density in the *ortho* position of the phenol promotes electrophilic attack on the flavin hydroperoxide [126]. However, there is considerable amount of doubt as to whether the enzyme actually achieves phenol deprotonation. It has been determined that with the formation of the hydroperoxyflavin that the substrate is protonated and that it is unfavorable for Asp54 to deprotonate the target substrate after formation of this intermediate [131]. Transient kinetic approaches tested this model using the mutant Asp54Asn, for which it was observed that the formation of peroxyflavin was significantly slower but the hydroxylation step was not affected suggesting that Asp54 was not responsible for deprotonation [93]. The mutant Tyr389Phe also showed little change to the oxidative reaction rate constants and therefore it also was not thought to be cooperating in deprotonation of substrate. Since these are the only two residues clearly ligand to the substrate this would suggest that deprotonation of the substrate by an adjacent residue does not occur. In addition, the hydroxylation reaction in PHHY is not influenced significantly by pH as it is with PHBH [110,132]. Therefore, the only means for substrate deprotonation could be between the substrate and the flavin peroxide. Much speculation over the deprotonation issue has surfaced and little of it has been resolved [93,120,131,133]. If deprotonation doesn’t take place, as the most recent data would suggest, it makes intuitive sense that this enzyme is less discriminating for which aromatic substrates it hydroxylates. For PHBH, discrimination for target substrates is based heavily on specific substituents, to the point that many catalytic steps are contingent on normal functioning of the hydrogen-bond chain that originates at the target substrate.

Whether the substrate is deprotonated or not in PHHY, the aromatic substrate *ortho* position is relatively nucleophilic and is attacked by the *C*(4a)-hydroperoxyflavin, cleaving the peroxy-bond. The transition state species seems to be stabilized by a hydrogen-bond formed between the carbonyl oxygen of Pro364 and the hydrogen on the flavin hydroperoxide [131]. As stated earlier, The Pro364Ser variant resulted in a more flexible *re*-side polypeptide. When either phenol or *m*-cresol were used as substrates, hydroxylation was disrupted, the enzyme achieving only ~60% hydroperoxyflavin formation and only ~20% target substrate hydroxylation. However, when resorcinol was used as substrate no significant change in hydroxylation from wild-type PHHY occurred suggesting that the doubly activated substrate is intrinsically more facile to hydroxylate [120].

No studies have been conducted to elucidate the mechanism of the oxidative half reaction for PgaE or CabE. However, as the model enzymes have illustrated above, deprotonation of the substrate prior to electrophilic attack by the hydroperoxyflavin is thought to be favorable for hydroxylation. However, neither PgaE or CabE possess a residue in close proximity to the substrate that could act as a base to deprotonate the ligand. Therefore, it is postulated that two scenarios could be at play, either the carbon atom at the site of hydroxylation is sufficiently nucleophilic to initiate hydroxylation or a keto-enol tautomerization could take place nearby on the substrate (C(12)) and donation of the hydrogen to the flavin peroxide could lead to increased charged density on the site of hydroxylation [7].

#### 2.2.4. Product Release

The decay of *C*(4a)-hydroxyflavin to regenerate the oxidized catalyst occurs with the release of product and water from the active site. These two steps are indistinguishable in kinetic experiments with WT PHBH in catalysis with the native substrate. The steps correspond to a first order reaction and are heavily dependent upon pH [99,125]. When hydroxylation is complete, it has been suggested that the hydrogen-bond network is utilized again for deprotonation of the product 4-OH and concomitant formation of a strong hydrogen-bond to Pro293 resulting in a dianion that may initiate the flavin conformational change necessary for expulsion of the product [84,125]. The indirect link between Arg214 and Arg44 of the *si*-face polypeptide, by way of the product, is disrupted causing a rotation of the *si*-face polypeptide to an unstrained position. This disruption is thought to occur by the strong hydrogen bond formed between 3,4DOHB to Pro293 and the rotation of the product within the active site (by 14°), as shown in the crystal structure of the enzyme-product complex [87,123]. The unstrained *si*-face peptide helps to establish the flavin *open* conformation that exposes the active site to solvent [67,72]. In the presence of solvent the *C*(4a)-hydroxyflavin readily decays to form water and regenerate the oxidized isoalloxazine [72]. It is thought that the product is first to leave as the presence of excess substrate can delay the decay of hydroxyflavin, presumably trapped by the substrate after the departure of the product [71,78]. This order of decay is not absolute however as the enzyme kynurenine 3-monooxygenase has been shown to possess a unique characteristic where the *C*(4a)-hydroxyflavin is not observed and seems to decay rapidly prior to product release, presumably due to a solvent molecule being in close proximity to the flavin in the product complex [82].

## 3. Perspective

The FPMO enzymes have a core catalytic purpose, to deliver one oxygen atom from dioxygen to a typically activated target substrate using a flavin cofactor and reducing equivalents from NAD(P)H. Numerous researchers have investigated the structure and mechanism of FPMO enzymes and these data have shown that chemistry catalyzed has considerable mechanistic commonality for individual catalytic steps. The majority of the research record describes one or two examples of the Class A FPMO, as these enzymes have proven to be the most amenable to a more intricate and definitive set of observational strategies. The extraordinary detail that has been obtained from the data amassed for *para*-hydroxbenzoate hydroxylase and phenol hydroxylase is clearly insufficient as a description for the FPMO enzymes as a whole and does not offer applicable mechanistic consensus for even the Class A members. Moreover, the breadth in the range of catalytic adaptations within FPMOs that conform the catalytic purpose to a given target substrate is sufficient to undermine any one enzyme acting as the class or family paradigm. The apparent myriad of gating strategies that require cofactor and/or peptide dynamics result in even closely grouped members of a single class exhibiting diversity in both the sequence of events and the molecular machinery that is mobilized to accomplish the chemistry. One may conclude that the chemistry of the FPMOs is largely known, but the means by which it is accomplished by individual members remains an exceedingly rich area of investigation.

## Abbreviations

FPMO: Flavoprotein monooxygenase
PHBH: para-hydroxybenzoate hydroxylase
MHBH: meta-hydroxybenzoate hydroxylase
PHHY: phenol hydroxylase
DHPH: dihydroxypyridine hydroxylase
PhzS: phenzine decarboxylase
CabE and PgaE: Flavoenzymes involved in the biosythesis of angucycline
RebC: rebeccamycin
FAD: flavin adenine dinucleotide
FMN: flavin mononucleotide
NADH: nicotinamide adenine dinucleotide
NADPH: nicotinamide adenine dinucleotide phosphate
pOHB: para-hydroxybenzoate
